# Civic Engagement and Social Connectedness in Rural Communities: The Role of Sociodemographic Factors and Social Determinants of Health in Rural Areas of the United States

**DOI:** 10.3390/socsci14110674

**Published:** 2025-11-19

**Authors:** Emma C. Lewis, Galen D. Eldridge, Deyaun L. Villarreal, Meredith L. Graham, Johanna Y. Andrews Trevino, Sara C. Folta, Jay E. Maddock, Meg S. Patterson, Elena Andreyeva, Rebecca A. Seguin-Fowler

**Affiliations:** 1 Institute for Advancing Health Through Agriculture, Texas A&M AgriLife Research, 17360 Coit Rd., Dallas, TX 75252, USA; 2 Friedman School of Nutrition Science and Policy, Tufts University, Boston, MA 02111, USA; 3 School of Public Health, Texas A&M University, College Station, TX 77843, USA; 4 Institute for Advancing Health Through Agriculture, Texas A&M AgriLife Research, Norman E. Borlaug Building, 498 Olsen Blvd, College Station, TX 77843, USA

**Keywords:** civic engagement, social connectedness, sociodemographic, social determinants of health, rural

## Abstract

This study examined whether civic engagement (CE) and social connectedness (SC) differ by sociodemographic characteristics and social determinants of health (SDOH). Baseline data were drawn from a rural community-randomized controlled trial (n = 2381). Sociodemographic characteristics included sex, age, race/ethnicity, marital status, education, employment, and income. SDOH measures included food insecurity, having a regular healthcare provider, housing instability, utility shutoffs, transportation access, and government assistance. CE measures included attitudes, behaviors, and mobilization, while SC measures included community health investment, social cohesion, and social networks. Bivariate associations were estimated using linear regression to assess relationships between CE and SC measures and sociodemographic and SDOH measures. Being married, college-educated, or employed were positively associated with multiple CE measures. SC measures were consistently higher among participants with greater educational attainment and lower among those experiencing food insecurity. Findings highlight persistent inequities in CE and SC across sociodemographic and SDOH factors within rural communities.

## 1. Introduction

Aspects of both civic engagement and social connectedness have been linked to improved individual health and wellbeing, with civic engagement linked to better psychological, physical, and mental health (Dubowitz et al. 2020) and strong social connections shown to play a crucial role in physical and mental health (Anderson et al. 2006; Anderson et al. 2007; Annesi 2011; Greaves et al. 2011; Jung and Brawley 2013; Schwarzer and Leppin 1991; Sheats et al. 2017; United States Institute of Peace n.d.; Wing et al. 2006). Understanding and promoting civic engagement and social connectedness is especially important in medically underserved rural areas, where geographic distance can contribute to perceived social isolation (Henning-Smith et al. 2018) and may limit access to healthcare clinics and resources (Rural Health Information Hub 2025). Additionally, sociodemographic factors and social determinants of health (SDOH) (e.g., employment, education, income, health insurance, food security) are consistently lower in rural areas compared to urban areas (Bhuiyan 2020). Residents of rural areas are also more likely to experience negative health outcomes, including poorer mental health, compared to their urban counterparts (Morales et al. 2020).

### 1.1. Civic Engagement

Increased civic engagement, or how “an active citizen participates in the life of a community in order to improve conditions for others or to help shape the community’s future” (Adler and Goggin 2005), can strengthen democracy, foster community involvement, and promote positive social change (Arvanitidis 2017). Civic engagement can improve health by affecting behavior change at each level of the socioecological model (i.e., individual, social, community/environment/policy) (McLeroy et al. 1988).

Promising evidence suggests civic engagement initiatives can result in positive behavior change (Brown et al. 2017; Cohen et al. 2013; Slater et al. 2016; Varma et al. 2015; Varma et al. 2016). For example, physical activity increased following an initiative in which local groups of residents used a $4000 stipend to implement a park-based activity program (Cohen et al. 2013). Another civic engagement project aimed at improving the built environment led to improved individual health outcomes, despite lacking a formal behavioral intervention (Brown et al. 2017). Civic engagement can also reduce health inequities by empowering underrepresented and marginalized groups to change policies, systems, and environments in their communities (Ford Foundation n.d.; Morales-Garzón et al. 2023). Community-driven development or change requires a triumvirate of mobilization, assets, and distribution (Okoth-Okelloh Abel and Mark 2013). Mobilization, encompassing human capital, social assets, self-efficacy, motivation, and individual participation (Jakes and Shannon 2002), enables residents to shift from observing their environment to actively improving it.

There is conflicting information regarding how civic engagement varies by sociodemographic characteristics (e.g., gender, age, income, education, race/ethnicity) and SDOH (e.g., food security). For example, older adults may be more likely to participate in civic engagement due to having deeper community roots, whereas younger people may be more involved in cause-oriented social activism (Arvanitidis 2017). People with lower incomes are generally less civically engaged in behaviors (e.g., voting, volunteering) and attitudes (e.g., satisfaction with politics), though this is likely driven by systemic and structural barriers that disproportionately affect low-income communities (Garon and Stacy 2024; McBride et al. 2006). Those struggling to meet basic needs (e.g., food, housing, healthcare, childcare) may deprioritize civic engagement in favor of survival-oriented activities (Food Assistance Match 2024). Yet, some studies have found that disposable income is not associated with civic engagement participation (Arvanitidis 2017).

Education appears strongly linked to civic engagement: individuals with college degrees are four to five times more likely to engage in civic actions than those without a high school diploma (National Conference on Citizenship n.d.). Higher education may equip individuals with the skills and confidence necessary to navigate civic institutions and social systems effectively. Patterns also differ by sex, with men less likely than women to utilize or engage with community and healthcare services, often due to cultural norms around masculinity and stigma (Kwon et al. 2023). Emerging evidence suggests these dynamics extend beyond healthcare; a recent study in Spain (Sánchez-García et al. 2024) found that women scored significantly higher than men in civic awareness and participation. Relatively few studies have examined civic engagement among racial and ethnic minority groups, and those that have often rely on non-representative samples (Phan and Kloos 2023). In general, perceived discrimination is associated with greater civic engagement and mobilization towards community change (Schildkraut and Mistry n.d.). Regarding geography, United States rural residents exhibit relatively high civic engagement compared to urban residents, potentially due to longer community tenure, lower population density, larger social networks, and greater community homogeneity (Thompson 2021). Despite this, we found no U.S. studies examining individual mobilization for community change by sociodemographic or SDOH characteristics. Thus, determining how civic engagement attitudes, behaviors, and mobilization differ across key characteristics and social factors remains challenging due to limited published research.

### 1.2. Social Connectedness

Social networks offer informational, functional, psychological, and social support (Hussain et al. 2023). A culture of social connectedness is one in which “good health and well-being flourish across geographic, demographic, and social sectors; fostering healthy equitable communities guides public and private decision making; and everyone has the opportunity to make choices that lead to healthy lifestyles” (Robert Wood Johnson Foundation n.d.). Levels of cultural and social connectedness may differ by sociodemographic characteristics (e.g., age, gender, income, race, ethnicity) and SDOH factors (Kannan and Veazie 2023).

Social connectedness refers to active participation in social roles, relationships, and activities. It is associated with larger and more diverse social networks, greater frequency and quality of social interactions, and more robust support systems, all of which positively influence health and longevity (Hamlin et al. 2022). For example, a longitudinal cohort study of African American participants found that higher social activity was associated with a 34% lower risk of premature mortality (Lamar et al. 2022). A study conducted among older women in Pittsburgh found that sustained participation in senior center activities was associated with fewer physical limitations and chronic conditions; notably, non-white participants participated at higher intensities (e.g., attend more events, spend more time per week with senior center activities) than white participants (Tang et al. 2011).

As with civic engagement, social connectedness is shaped by various sociodemographic factors such as age, race, ethnicity, socioeconomic status, and geography. Older adults often report more positive and satisfying social relationships (Luong et al. 2011). In some cultural contexts, men may have greater access to social activities than women (Ong et al. 2024). A systematic review found that higher socioeconomic status was consistently associated with increased participation in social activities among older adults (Ong et al. 2024). In another study, non-Hispanic Black participants engaged in fewer social activities than non-Hispanic white participants despite similar network size and support levels (Hamlin et al. 2022). Lower socioeconomic status was also associated with higher loneliness, social isolation, and lack of support in a large United Kingdom sample (Kung et al. 2022). Social participation may also differ by geography; older rural adults tend to be less socially active than their urban counterparts (Vogelsang 2016).

Social cohesion, defined as “the ongoing process of developing well-being, sense of belonging, and voluntary social participation of the member of society, while developing communities that tolerate and promote a multiplicity of values and cultures, and granting at the same time equal rights and opportunities in society” (Moustakas 2023), also varies by sociodemographic factors and SDOH. For example, lower neighborhood social cohesion was associated with higher odds of food insecurity in a large sample of California women with young children, even after controlling for household socioeconomic factors (Denney et al. 2017). A nationwide sample of 167,000 adults found that younger adults (aged 18–30 years old) and non-Hispanic Black adults were more likely to live in neighborhoods with low social cohesion (Alhasan et al. 2020). Community investment in health is typically measured by assessing investment in resources that promote health and wellbeing (Chandra et al. 2017). Few surveys measure this construct directly (Carman et al. 2019), limiting understanding of variation across sociodemographic and SDOH factors. In the 2018 National Survey of Health Attitudes, 28% of respondents did not consider community health investment a top priority (Carman et al. 2019). However, those who did were more likely to engage civically to address health issues (Dubowitz et al. 2020).

Civic engagement and social connectedness intersect with multiple social and economic domains that directly influence health at individual, household, and community levels. Given their potential to inform research, program development, and policy decisions—and in light of elevated morbidity and mortality rates in rural communities nationwide—it is in the interest of public, private, and research sectors to rigorously measure and examine how these factors vary across sociodemographic groups and SDOH (Habermann et al. 2014). To address this need, we analyzed data from a sample of residents enrolled in a community trial conducted in medically underserved rural areas to evaluate whether civic engagement and social connectedness varied by sociodemographic characteristics and SDOH. As a first step toward this objective, we assessed civic engagement (attitudes, behaviors, and mobilization) and social connectedness (social network, social cohesion, and community investment in health). These descriptive analyses provided foundational insight into potential disparities in civic engagement and social connectedness across key demographic and social determinant factors.

## 2. Materials and Methods

This study used a cross-sectional design to explore associations between civic engagement and social connectedness measures and various sociodemographic and SDOH variables among rural adults. The sample was part of a community-randomized controlled intervention trial that was designed to use civic engagement to promote small community changes to improve healthy eating and physical activity. The present analyses are based on baseline data collected between 2022–2023. Although data were derived from a randomized trial, they do not assess intervention effect. Data from all sample members were included (n = 2381). Sample members voluntarily enrolled in the randomized trial in 12 rural towns in New York and Texas. The lead investigators of the study were based in New York and Texas and had existing connections with the Extension systems in those states that led the implementation, which was essential for study execution. Prior to the start of the project, Extension educators in qualifying communities were contacted, discussed the project with the lead investigators, reviewed the scope of work for the project, and, if interested, signed on to participate. Inclusion criteria were being 18 years of age or older, considering themselves a member of one of the 12 study communities, and having a valid email address. Communities were defined by between 1 and 8 ZIP Code Tabulation Areas, and all eligible ZIP codes for each community were targeted for social media advertising, postcards, and letters. Recruitment approaches included online advertisements and social media posts, postal mailings and emails, word-of-mouth referrals, public events, flyers/posters, and radio/television stories and advertisements. All baseline data were obtained via an online questionnaire.

### 2.1. Measures

#### 2.1.1. Sociodemographic Characteristics

Sociodemographic characteristics included sex, age, race/ethnicity, marital status, college graduate, employment status, and annual household income (all dollar amounts are presented in USD). A cut-off of $50,000 was chosen because studies indicate that a larger percentage of households in rural areas fall below the $50,000 income threshold compared to non-rural areas (Ramakrishnan and Suandi 2024); a lower household income, particularly below a threshold like $50,000, has been linked to various social and health disparities (Alsaiari et al. 2024) and 49.7% of the sample had an annual household income less than $50,000 per year.

#### 2.1.2. Social Determinants of Health Measures

Measures of SDOH included experiencing household food insecurity in the last 12 months (Gundersen et al. 2017), not currently having a regular healthcare provider (Gadhoke et al. 2018), current housing situation (Billioux et al. 2017), having utilities shut off in the last 12 months (Billioux et al. 2017), lacking access to transportation in the last 12 months (Billioux et al. 2017), and receiving any government assistance (including Special Supplemental Nutrition Program for Women, Infants, and Children, Supplemental Nutrition Assistance Program, Temporary Assistance for Needy Families, Supplemental Security Income, Social Security Disability, or General Assistance, but not including social security benefits) at the time of the survey.

#### 2.1.3. Civic Engagement Measures

Civic engagement measures are described here, and full questionnaires are available as Supplementary Materials to this article. Measures of civic engagement included civic engagement attitudes, behaviors, and mobilization.

Civic engagement attitudes and behaviors were measured using Doolittle and Faul’s Civic Engagement Scale (Doolittle and Faul 2013). The attitudes subscale contained eight statements, which were responded to using a 5-point Likert scale from 1 = Strongly Disagree to 5 = Strongly Agree. Example statements include “I feel responsible for my community,” “I believe that it is important to be informed of community issues,” and “I am committed to serving in my community.” The eight items were averaged to create an overall attitudes score (range: 1–5). The behaviors subscale contained six statements which were responded to using a 5-point scale from 1 = Never to 5 = Always. Example statements include “I am involved in regular volunteer position(s) in my community,” “I help members of my community,” and “I stay informed of events in my community.” The six items were averaged to create an overall civic engagement behaviors score (range: 1–5).

Mobilization was measured using Jakes and Shannon’s Mobilization Scale (Jakes and Shannon 2002), which consists of eight statements answered on a 5-point Likert scale from 1 = Strongly Disagree to 5 = Strongly Agree. Example statements include “I know how to work with others to solve problems,” “I have the communication skills to influence people in my community,” and “I know how to raise money to do community action projects.” Two items were reverse coded, and then the eight items were averaged to create a mobilization score (range: 1–5).

#### 2.1.4. Social Connectedness Measures

Measures of social connectedness included investment in community health, community cohesion, and social networks. The full questionnaires are available as Supplementary Materials to this article.

Social networks were measured using the Lubben Social Network Scale (Lubben et al. 2006). For family, there were three questions, which began with the instructions “Considering the people to whom you are related by birth, marriage, adoption, etc.” and included three questions “How many relatives do you see or hear from at least once a month?”, “How many relatives do you feel at ease with that you can talk about private matters?”, and “How many relatives do you feel close to such that you could call on them for help?” For friends, the three questions were the same, but began with the phrase “How many of your friends…”. Response options for all six questions were 0 = 0, 1 = 1, 2 = 2, 3 = 3 or 4, 4 = 5–8, 5 = 9 or more. Responses to the six items were summed to create an overall social network score (range: 0–30). That score was then divided by 6 for an analytic score ranging from 0 to 5.

Social cohesion was measured using Mujahid et al.’s 4-item Neighborhood Environment Scale (Mujahid et al. 2007), which uses a 5-point Likert scale from 1 = Strongly Disagree to 5 = Strongly Agree. Sample statements include “People in my community are willing to help their neighbors” and “People in my community generally get along with each other.” Responses were averaged to create a social cohesion score (range: 1–5).

Investment in community health was measured via the 5-item Robert Wood Johnson Foundation National Survey of Health Attitudes subscale (Carman et al. 2019) with response options ranging from 1 = Very high priority to 5 = Not a priority at all. The instructions for this section were as follows: “In the following section, we list goals that some people think are important for communities in the U.S. In these statements, when we refer to “communities,” we mean all communities, not just your own. Should the following be a ‘very high priority’, ‘high priority’, ‘important but not a top priority’, ‘low priority’, or ‘not a priority at all’ for communities?” Example statements include “Making sure that the disadvantaged have an equal opportunity to be healthy,” “Making sure that healthy foods are for sale at affordable prices in communities with limited access,” and “Making sure that there is decent housing available for everyone who needs it.” The number of items ranked as 1 = very high priority or 2=high priority was calculated and categorized as follows: 0 = no high priorities, 1 = one high priority, 2 = two high priorities, 3 = three high priorities, 4 = four high priorities, 5 = five high priorities. These categories comprised an investment in community health score (range: 0–5).

### 2.2. Statistical Analysis

Descriptive statistics for sample characteristics and measures of civic engagement and social connectedness were tabulated (Tables 1 and 2). To assess variability across the scales, coefficients of variation were calculated for each scale by diving the standard deviation by the mean (Table 2). We assessed the normality of all measures and found all variables to have skewness values less than 2 (George and Mallery 2016). Linear regression was used to examine bivariate associations between (1) civic engagement measures and sociodemographic and SDOH measures, and (2) social connectedness measures and sociodemographic and SDOH measures (Table 3). Race and ethnicity were modeled jointly in multivariate regression (variables of Black non-Hispanic, Hispanic, and Others) with white non-Hispanic as the reference group. Tests were considered significant at 95% confidence or higher after Bonferroni correction to account for three measures of each construct (civic engagement and social connectedness). Analyses were performed using SPSS version 29 (IBM Corp., Armonk, NY, USA).

### 2.3. Ethics Approval

The study was approved by the Texas A&M University Human Subjects Protection Program via full review, and written (online) consent was obtained (protocol # IRB2021-1490).

## 3. Results

The majority of the sample was female (67.7%), less than 65 years old (86.2%), and non-Hispanic white (85.8%) (see Table 1). About half of the sample was married (53.4%), 47.4% had a college degree or more education, 63.6% were employed or self-employed, and 49.7% had an annual household income of less than $50,000. About one-third of the sample had experienced food-insecurity in the past year (39.6%). The majority owned a home or apartment (63.6%). Less than a quarter of the sample did not have a regular healthcare provider (12.9%), had their utilities shut off or threatened (13.6%), or lacked transportation (15.0%) in the last 12 months, while 22.8% received government assistance.

Average scores for civic engagement factors (on a scale of 1–5) ranged between 3.00 and 3.74 (see Table 2) with moderate variability. The average score for investment in community health (range: 0–5) was 3.92 with SD = 1.51. Social cohesion had a mean of 2.53 (SD = 0.75), while social network averaged 3.50 (SD = 0.95); both were measured on a 1–5 scale. The coefficients of variation for civic engagement attitudes, mobilization, and social network were less than 20%. However, the coefficients of variation for civic engagement behavior, investment in community health, and social cohesion were greater than 30%.

### 3.1. Associations with Civic Engagement

Table 3 presents the associations between sociodemographic characteristics and SDOH measures with various civic engagement measures.

#### 3.1.1. Associations Between Sociodemographic Characteristics and Civic Engagement Measures

Sociodemographic characteristics were sometimes associated with civic engagement measures (see Table 3). Being married, having a college degree or more education, and being employed were positively associated with all measures of civic engagement. An annual household income under $50,000 was negatively associated with all measures of civic engagement. Being male was negatively associated with civic engagement attitudes while being Black and non-Hispanic was positively associated with civic engagement behaviors and mobilization.

#### 3.1.2. Associations Between Social Determinants of Health Measures and Civic Engagement Measures

SDOH measures were sometimes associated with civic engagement measures. Not having a regular healthcare provider was negatively associated with all measures of civic engagement. Owning a home or apartment was positively associated with civic engagement attitudes and behaviors. Experiencing food insecurity in the past year was associated with lower levels of civic engagement attitudes and behaviors. Receiving any government assistance was only negatively associated with civic engagement behaviors.

### 3.2. Associations with Social Connectedness

Associations between sociodemographic characteristics and SDOH measures with social connectedness measures are described below (see Table 3).

#### 3.2.1. Associations Between Sociodemographic Characteristics and Social Connectedness Measures

Sociodemographic characteristics were sometimes associated with social connectedness (see Table 3). Having a college degree or more education was positively associated with all social connectedness measures. Being married was positively associated with social cohesion and network and being employed was only positively associated with social network. Being 65 years and older was positively associated with social cohesion and network. Being male was negatively associated with investment in community health and being Black non-Hispanic and other race and ethnicities were negatively associated with social network while other race and ethnicities was negatively associated with social cohesion. An annual household income under $50,000 was negatively associated with community social cohesion and network.

#### 3.2.2. Associations Between Social Determinants of Health and Social Connectedness Measures

SDOH were frequently associated with social connectedness. Experiencing food insecurity in the past year was negatively associated with all social connectedness measures. Not having a regular healthcare provider was negatively associated with social cohesion and network. Owning a home or apartment was negatively associated with investment in community health but positively associated with community social cohesion and network. Utilities shut off or threatened, lack of transportation, and receiving government assistance were all negatively associated with social cohesion and network, but receipt of government assistance was positively associated with investment in community health.

## 4. Discussion

Among a predominantly older, female, and white sample of adults living in rural Texas and New York communities, we found compelling associations between sociodemographic characteristics and civic engagement and social connectedness, as well as between SDOH and these outcomes. Study communities were sociodemographically similar to national and state-level rural populations, with a few exceptions related to race and ethnicity (Seguin-Fowler et al. forthcoming). Likewise, study participants were relatively representative of their respective community populations across most characteristics (Seguin-Fowler et al. forthcoming).

### 4.1. Sociodemographic Associations with Civic Engagement and Social Connectedness

Among sociodemographic characteristics, being male was negatively associated with both civic engagement and social connectedness. This finding aligns with the substantial body of research demonstrating that men are less likely than women to engage with community and healthcare services, particularly those perceived as care-oriented or relational (Kwon et al. 2023). The authors of this study posit that this could be explained by the Social Role Theory, in which women are more likely to care about issues related to care tasks, community aid, and voting in elections (Sánchez-García et al. 2024). Conversely, men may not only feel less drawn to civic and social activities but may also perceive fewer opportunities to participate in them. This has important implications, particularly as civic disengagement can contribute to broader feelings of social isolation or disconnection. Notably, another study found that civic engagement was positively associated with wellbeing for both men and women, with self-efficacy and meaning in life serving as mediators (Fenn et al. 2021). This suggests that although men may engage less frequently in civic and social activities, increasing access to meaningful and identity-congruent forms of engagement could offer important mental and emotional benefits. Therefore, recognizing and addressing gendered barriers to civic engagement and social connectedness could be a critical step toward fostering greater inclusion and overall wellbeing, especially in settings such as rural communities where traditional notions of masculinity may be more present (Silva 2022).

Having an annual household income of less than $50,000 was also negatively associated with both civic engagement and social connectedness. This finding is consistent with prior research demonstrating that people with lower incomes are generally less engaged civically than the population at large. As mentioned, this may be due to systemic and structural barriers that disproportionately affect low-income communities. In fact, studies have found that people with lower incomes face significant barriers in participating in civic and social activities due to limited access to transportation, inflexible work schedules, and unmet childcare needs (Lee 2025). These barriers can be especially pronounced in rural communities, where residents may face long travel distances to polling places, community meetings, or public forums, and may have fewer accessible channels for voicing concerns or opinions (Eisenberg 2022; Secure Democracy USA 2022). However, civic programs and community-based initiatives often underestimate the time, effort, and resources needed to reach and retain low-income citizens in participatory policy and social action work (De Weger et al. 2022). In other words, there is potential for increasing the civic capacity of lower income community members given a better understanding of how social factors related to wealth shape their needs.

Interestingly, in our relatively homogeneous sample, belonging to a racial and ethnic minority group was positively associated with civic engagement but negatively associated with social network and social cohesion. This divergence may reflect a complexity related to navigating civic and social life in the context of racialized social experiences. As mentioned, few studies have examined where racial and ethnic minority groups fall along the continuum of civic engagement (Phan and Kloos 2023). That said, our findings generally reflect the existing studies that have found perceived discrimination to be associated with greater levels of civic engagement, activism, and mobilization towards community change (Schildkraut and Mistry n.d.). In this context, civic engagement may serve as a form of resistance, empowerment, and collective coping. At the same time, other studies have shown that chronic exposure to racism and systemic inequities can erode trust in public institutions and lead to diminished engagement in civic structures perceived as unwelcoming or unjust (Tran et al. 2024). Related to social connectedness, another study also found lower social connectedness among racial and ethnic minorities, where more non-Hispanic Black and Hispanic adults lived in a neighborhood with low (compared to medium or high) social cohesion compared to non-Hispanic white adults (Alhasan et al. 2020). This paints a complex picture, in which perceived discrimination may simultaneously motivate individuals to work toward social change while also weakening social ties and alienating individuals and communities from larger social and political systems. Distinguishing between types of engagement and understanding how social characteristics and determinants shape patterns of participation remains crucial for communities that have historically experienced marginalization and civic exclusion.

Regarding other individual-level characteristics, we found that being married, having a college degree, and being employed were positively associated with both civic engagement and social connectedness, while being older was positively associated only with social connectedness. These findings are consistent with broader literature—for example, data from the General Social Survey (2012–2014) show that married adults are more likely to do volunteer work than their single counterparts, and marriage increased the likelihood of volunteering by 10 percentage points (Wolfinger 2019). Education has also emerged as a predictor of both civic engagement and social networking. Previous research found that adults with a higher education tended to report better health and wellbeing, were more likely to have fulfilling jobs, and were more civically engaged (Lumina Foundation 2023). College graduates have been shown to be significantly more likely to be involved in their communities, whether through volunteering, voting, or other forms of civic participation (National Conference on Citizenship n.d.). Similarly, employment provides financial stability as well as opportunities for social interaction and a greater sense of purpose, all of which contribute to social connectedness. Being employed can also create opportunities for civic engagement through workplace initiatives and networking. Some workplaces encourage civic participation directly, through volunteer days, donation drives, or partnerships with community organizations. These patterns underscore the role of individual-level resources in fostering civic engagement and social wellbeing and may be especially important to consider in rural areas where educational attainment and employment outcomes tend to be lower than in urban areas.

In terms of age, previous research indicates that being older is often linked to more positive and satisfying social relationships (Luong et al. 2011). One explanation for these findings is rooted in Socioemotional Selectivity Theory, which suggests that as people age, they become more selective in their social networks, prioritizing emotionally meaningful relationships rather than expanding their social circles (Carstensen 2021). Another explanation is that older people may have deeper community roots (Arvanitidis 2017). Additionally, older adults often benefit from reduced social demands (e.g., childrearing, work-related responsibilities) which may allow them to allocate more time and energy to nurturing supportive connections. Over the life-course, adults also tend to develop greater interpersonal skills, which can contribute to more enriching social interactions. These social strengths not only foster better relationship quality but also contribute directly to physical, mental, and social health. For example, Cornwell et al. (2008) posited that community involvement and civic engagement contribute to successful aging (Cornwell et al. 2008). For older adults living in rural communities, recognizing and leveraging social assets could be a vital strategy for enhancing community cohesion and promoting overall wellbeing across the lifespan.

### 4.2. Social Determinants of Health Associations with Civic Engagement and Social Connectedness

Among our sample, we also observed a wide range of associations between key SDOH and both civic engagement and social connectedness, underscoring how social conditions influence individuals’ capacity and desire to participate in their communities. For example, experiencing food insecurity within the past year was negatively associated with civic engagement attitudes and behaviors, as well as all measures of social connectedness. Previous research on food insecurity, social capital, and reliance on social support is complex and sometimes mixed, with some studies suggesting that individuals struggling to meet basic needs may deprioritize both civic participation and social interaction in favor of survival-oriented activities (Food Assistance Match 2024). Food insecurity can also be a cause for social isolation, limiting individuals’ ability to participate fully in society due to financial and interpersonal constraints (Community Food Centres 2021). On the other hand, individuals may rely more heavily on social networks and social capital to cope with food insecurity (Nosratabadi et al. 2020). It is important to note, however, that the latter typically occurs within more informal social networks when formal civic and social resources are otherwise lacking. Interestingly, Leddy et al. (2020) found differences in food-insecure women’s use of bonding social capital (i.e., depending on support from members within one’s own social group) compared to bridging (i.e., building relationships with members from other social groups) and linking (i.e., building relationships with representatives of institutions) social capital, for gaining access to food (Leddy et al. 2020).

Not having a regular healthcare provider was a consistent negative predictor across all outcomes including civic engagement, social cohesion, and social networking. Access to routine care may facilitate trust in institutions, improve health literacy, and serve as a point of connection to community resources and networks (National Academies of Sciences, Engineering, and Medicine 2023). In rural communities where healthcare access is often limited (Rural Health Information Hub 2025), this finding highlights the broader role of healthcare providers as not just clinicians but potential gateways to civic and social participation. In fact, Habib et al. (2023) argue that health and civic engagement are reciprocally and longitudinally linked such that a robust primary care system has the potential to facilitate increased civic engagement (e.g., voting, volunteerism, community service, political involvement) at the community level (Habib et al. 2023). The authors call out providers to share the responsibility for promoting both health and civic engagement with educators, policymakers, organizations, and larger systems on a whole.

In addition, adults in our sample who owned their home or apartment were more likely to report positive civic engagement attitudes and behaviors, and greater community social cohesion and engagement, which is consistent with the idea that residential stability fosters long-term investment in local networks. This is further confirmed by research conducted as part of the National Association of Realtors, which demonstrates that homeowners move less frequently than renters, and therefore become embedded into the same neighborhood and community for longer periods of time (National Association of Realtors^®^ 2012). However, we found that home ownership was negatively associated with investment in community health, which could suggest that those with more secure housing may perceive less urgency for improving the broader conditions of their communities. It is possible this could be tied to neighborhood context—those renting in multi-unit buildings are more likely to be surrounded by immediate social disorder and crime whereas homeowners may be further removed from safety concerns (Swope and Hernández 2019). In rural areas, where homeownership is often tied to generational landholding and place identity (Bonnie et al. 2020; Diamond 2023), homeownership may influence perceptions of responsibility for collective action and wellbeing.

Having utilities shut off or threatened and lacking reliable transportation were both negatively associated with community social cohesion and network. This also aligns with our findings regarding having lower incomes. Having financial difficulties is associated with lower feelings of belonging to society and the community, which could play into attitudes and behaviors of civic engagement (Villalonga-Olives et al. 2018). Research on utility costs has found that experiencing shut offs can lead to negative feelings of shame, anxiety, and depression, which in turn can lead to social isolation, reduced community ties, strained relationships, and disrupted social networks (Hernandez 2016). Similarly, utility insecurity may serve as a marker of chronic financial instability that can reduce capacity for both social interaction and civic involvement. Moreover, according to the Rural Health Information Hub, transportation is essential for civic engagement and participation in community life (Rural Health Information Hub n.d.), but rural transportation challenges are well documented.

Our results show that while individuals receiving government assistance were less likely to engage in civic behaviors, they demonstrated a greater investment in community health. Swartz et al. (2009) found that welfare recipients were less likely to vote than nonrecipients, but this did not hold true for recipients of non-means-tested government assistance (Swartz et al. 2009). These same authors discuss previous research wherein some scholars argue that the lower voting rates among welfare recipients can be explained by pre-existing characteristics associated with low voter turnout (e.g., being poorer, less educated, and younger) while others suggest that this relationship holds true regardless of sociodemographic characteristics (Swartz et al. 2009). For example, Bruch et al. (2010) found that recipients of Temporary Assistance for Needy Families (TANF) reported significantly less voting and civic participation even after controlling for social and demographic factors (Bruch et al. 2010). Concurrently, those receiving government assistance may still recognize the need for systemic improvements, leading to a desire to invest in their communities. This complexity is especially important to consider in rural areas where social services may be more fragmented and carry greater social stigma.

Taken together, our findings begin to demonstrate how various characteristics, such as being married, having a college degree, and being employed, can support civic engagement and social connectedness. In contrast, characteristics like experiencing food insecurity can hinder these outcomes, particularly in rural communities where residents often face greater inequities and isolation. Understanding and addressing individual and systemic social conditions is essential for designing interventions, programs, and policies that effectively and sustainably cultivate engaged, cohesive, and resilient communities. The present study has several strengths and limitations worth noting. One strength is that the analyses utilize data collected from a randomized trial with a relatively large sample size for a study population in medically underserved rural communities. However, our sample had relatively homogenous sociodemographic characteristics, which may limit generalizability to populations with other racial or ethnic identities. Another limitation was the potential for selection bias related to those who chose to participate in research; to address this, we used a multi-pronged recruitment strategy. Comparison of study participants to publicly available data for the relevant communities confirmed there were relatively few sociodemographic differences between our sample and broader communities (Seguin-Fowler et al. forthcoming). In addition, measures of civic engagement and social connectedness were collected via self-reported questionnaires, which may limit the reliability of our findings.

## 5. Conclusions

Each civic engagement and social connectedness measure varied across multiple sociodemographic characteristics or SDOH, indicating substantial variability in these outcomes within this population. Future work should explore whether these findings are generalizable to other populations, and future public health interventions should consider adapting their programs to promote civic engagement and social connectedness based on the populations they aim to reach.

## Supplementary Material

Supplementary Material

**Supplementary Materials:** The following supporting information can be downloaded at: https://www.mdpi.com/article/10.3390/socsci14110674/s1, Questionnaires for “Civic Engagement and Social Connectedness in Rural Communities: The Role of Sociodemographic Factors and Social Determinants of Health in rural areas of the United States”.

## Figures and Tables

**Table 1.** Sociodemographic characteristics of the sample and social determinants of health measures.

		Count	%
**Sociodemographic Characteristics**		**(n = 2381)**	
Sex	Male	766	32.2
	Female	1611	67.7
	Not one of the above	4	0.2
Age Group	18 to 24 years	170	7.1
	25 to 34 years	544	22.8
	35 to 44 years	544	22.8
	45 to 64 years	795	33.4
	65 years and older	328	13.8
Race	American Indian/Alaska Native	27	1.1
	Asian	26	1.1
	Native Hawaiian/Pacific Islander	5	0.2
	Black	169	7.1
	White	2044	85.8
	More than one race	75	3.1
	Not one of the above	35	1.5
Hispanic	Yes	205	8.6
Relationship Status	Married	1271	53.4
	A member of an unmarried couple	270	11.3
	Divorced	276	11.6
	Widowed	80	3.4
	Separated	57	2.4
	Never been married	427	17.9
Highest year of school	High school or less	606	25.5
	Technical or vocational school	97	4.1
	Some college	550	23.1
	College graduate	737	31.0
	Graduate or professional degree	391	16.4
Employment status	Employed	1365	57.3
	Self-employed	149	6.3
	Out of work for 1+ years	65	2.7
	Out of work for <1 year	62	2.6
	Homemaker	132	5.5
	Student	76	3.2
	Retired	347	14.6
	Unable to work	185	7.8
Annual household income	Less than $20,000	474	19.9
	$20,000-$24,999	109	4.6
	$25,000-$34,999	229	9.6
	$35,000-$49,999	371	15.6
	$50,000-$74,999	474	19.9
	$75,000 or more	724	30.4
**Social determinants of health measures**			
Household experienced food insecurity in last 12 months		944	39.6
Do not have a regular healthcare provider		308	12.9
Current housing situation	Own a house or apartment	1514	63.6
	Rent entire house/apartment	622	26.1
	Rent a room in a house or apartment	75	3.1
	Public housing	34	1.4
	Staying with friends or family	120	5.0
	Unhoused, supportive housing, or shelter	16	<1%
Utilities shut off or threatened in last 12 months		325	13.6
Lack of transportation in last 12 months		356	15.0
Received any government assistance at time of survey		543	22.8

**Table 2.** Mean civic engagement and social connectedness measures.

	Mean (n = 2381)	Standard Deviation	Coefficient of Variance
**Civic Engagement**			
Civic engagement attitudes subscale^a^	3.74	0.63	16.8%
Civic engagement behaviors subscale^a^	3.00	0.91	30.3%
Mobilization scale^a^	3.33	0.65	19.5%
**Social Connectedness**			
Social network scale^b^	3.50	0.75	21.4%
Social cohesion scale^a^	2.53	0.95	37.5%
Investment in community health subscale^b^	3.92	1.51	38.5%

a Range: 1–5.

b Range: 0–5.

**Table 3.** Associations between sociodemographic characteristics and social determinants of health with civic engagement and social connectedness.

	Civic Engagement						Social Connectedness					
	Civic Engagement Attitudes (Range: 1–5)		Civic Engagement Behaviors (Range: 1–5)		Mobilization (Range: 1–5)		Social Network (Range: 0–5)		Social Cohesion (Range: 1–5)		Investment in Community Health (Range: 0–5)	
	B	95% CI	β	95% CI	β	95% CI	β	95% CI	β	95% CI	β	95% CI
**Sociodemographic characteristics**		
Male	**−0.09** *	**(−0.15, −0.02)**	−0.07	(−0.16, 0.03)	+0.04	(−0.03, 0.10)	−0.03	(−0.13, 0.07)	+0.07	(−0.01, +0.15)	**−0.51** *	**(−0.67, −0.36)**
65 and older	−0.02	(−0.11, 0.07)	+0.06	(−0.07, 0.19)	−0.05	(−0.14, 0.05)	**+0.15** *	**(0.01, 0.28)**	**+0.15** *	**(0.05, 0.26)**	−0.06	(−0.27, 0.16)
Race and ethnicity^a^												
White non-Hispanic (reference group)												
Black non-Hispanic	+0.04	(−0.09, 0.17)	**+0.23** *	**(0.05, 0.41)**	**+0.17** *	**(0.04, 0.30)**	**−0.26** *	**(−0.45, −0.08)**	−0.02	(−0.17, 0.13)	+0.19	(−0.10, 0.49)
**Sociodemographic characteristics**												
Hispanic	−0.00	(−0.12, 0.11)	−0.03	(−0.20, 0.14)	−0.00	(−0.13, 0.12)	−0.16	(−0.34, 0.02)	−0.06	(−0.08, 0.20)	−0.26	(−0.54, 0.08)
Others	−0.02	(−0.15, 0.11)	+0.03	(−0.15, 0.22)	+0.02	(−0.12, 0.15)	**−0.22** *	**(−0.42, −0.03)**	**−0.24** *	**(−0.39, −0.08)**	+0.27	(−0.04, 0.57)
Married	**+0.09** *	**(0.02, 0.15)**	**+0.17** *	**(0.08, 0.26)**	**+0.07** *	**(0.01, 0.13)**	**+0.23** *	**(0.13, 0.32)**	**+0.18** *	**(0.10, 0.25)**	−0.10	(−0.25, 0.05)
College graduate or more	**+0.22** *	**(0.16, 0.28)**	**+0.30** *	**(0.21, 0.39)**	**+0.17** *	**(0.11, 0.23)**	**+0.39** *	**(0.30, 0.48)**	**+0.20** *	**(0.12, 0.27)**	**+0.23** **	**(0.08, 0.37)**
Employed	**+0.09** *	**(0.02, 0.15)**	**+0.12** *	**(0.03, 0.21)**	**+0.11** *	**(0.05, 0.18)**	**+0.29** *	**(0.20, 0.39)**	+0.07	(−0.01, 0.15)	−0.10	(−0.26, 0.05)
Annual household income <$50,000	**−0.14** *	**(−0.20, −0.08)**	**−0.24** *	**(−0.33. −0.15)**	**−0.13** *	**(−0.19, −0.06)**	**−0.52** *	**(−0.61, −0.43)**	**−0.25** *	**(−0.33, −0.18)**	+0.06	(−0.09, 0.21)
**Social determinants of health**												
Household experienced food insecurity in last 12 months	**−0.03** *	**(−0.06, −0.00)**	**−0.06** *	**(−0.10, −0.02)**	−0.02	(−0.05, 0.01)	**−0.16** *	**(−0.20, −0.12)**	**−0.06** *	**(−0.09, −0.02)**	**−0.11** *	**(−0.18, −0.04)**
Do not have a regular healthcare provider	**−0.16** *	**(−0.07, −0.25)**	**−0.30** *	**(−0.17, −0.44)**	**−0.16** *	**(−0.07, −0.26)**	**−0.27** *	**(−0.13, −0.41)**	**−0.19** *	**(−0.08, −0.30)**	−0.14	(0.09, −0.36)
Own home or apartment	**+0.08** *	**(0.01, 0.14)**	**+0.13** *	**(0.04, 0.22)**	+0.01	(−0.06, 0.07)	**+0.38** *	**(0.28, 0.47)**	**+0.20** *	**(0.12, 0.27)**	**−0.18** *	**(−0.33, −0.03)**
Utilities shut off or threatened in last 12 months	+0.01	(−0.08, 0.10)	−0.07	(−0.20, 0.06)	+0.00	(−0.09, 0.10)	**−0.42** *	**(−0.55, −0.28)**	**−0.30** *	**(−0.41, −0.20)**	+0.02	(−0.20, 0.23)
Lack of transportation in last 12 months	−0.07	(−0.16, 0.02)	−0.03	(−0.16, 0.09)	−0.01	(−0.10, 0.08)	**−0.61** *	**(−0.74, −0.48)**	**−0.33** *	**(−0.43, −0.22)**	+0.00	(−0.21, 0.21)
Received government assistance at time of survey	−0.07	(−0.14, 0.01)	**−0.16** *	**(−0.27, −0.05)**	+0.01	(−0.07, 0.08)	**−0.51** *	**(−0.62, −0.40)**	**−0.20** *	**(−0.29, −0.11)**	**+0.25** *	**(0.07, 0.42)**

β and 95% confidence intervals are from bivariate linear regressions with Bonferroni correction. Bold and * indicates significant associations.

a Race and ethnicity were modeled simultaneously in multivariate regression.

## Data Availability

The data presented in this study are available upon reasonable request from the corresponding author.

## Abbreviations

CE: Civic engagement
SC: Social connectedness
SDOH: Social determinants of health
