# Supplementary material for: Civic Engagement and Social Connectedness in Rural Communities: The Role of Sociodemographic Factors and Social Determinants of Health in Rural Areas of the United States
Source: Soc Sci. Author manuscript; Available in PMC 2026 Jun 27. (PMC13309175; doi:10.3390/socsci14110674)
Supplement: Supplementary Material [file NIHMS2187610-supplement-Supplementary_Material.pdf]

Supplemental Material

Questionnaires for "Civic engagement and social connectedness in rural communities: the role of sociodemographic factors and social determinants of health in rural areas of the United States"

CIVIC ENGAGEMENT QUESTIONNAIRES

General civic engagement attitudes and behaviors

The following questions ask about your attitudes towards civic engagement. Civic attitudes represent individuals' personal beliefs about their own involvement in the community and their perceived ability to make a difference. Please indicate the extent to which you agree or disagree with each statement below.					
	Strongly Disagree	Disagree	Neither Agree Nor Disagree	Agree	Strongly Agree
1. I feel responsible for my community.	1	2	3	4	5
2. I believe I should make a difference in my community.	1	2	3	4	5
3. I believe that I have a responsibility to help those in need.	1	2	3	4	5
4. I am committed to serving in my community.	1	2	3	4	5
5. I believe that all people have a responsibility to their community.	1	2	3	4	5
6. I believe that it is important to be informed of community issues.	1	2	3	4	5
7. I believe that it is important to volunteer.	1	2	3	4	5
8. I believe that it is important to financially support charitable organizations.	1	2	3	4	5
Now we would like to ask about your civic engagement behaviors. Civic behaviors are the actions individuals take to actively engage and make a difference in their community. Please indicate the extent to which you have engaged in the following behaviors on a scale from never to always.					
	Never	Rarely	A few times	Often	Always
9. I am involved in regular volunteer position(s) in my community.	1	2	3	4	5

10. When working with others, I make positive changes in my community.	1	2	3	4	5
11. I help members of my community.	1	2	3	4	5
12. I stay informed of events in my community.	1	2	3	4	5
13. I participate in discussions that raise issues of social responsibility.	1	2	3	4	5
14. I contribute to charitable organizations within my community.	1	2	3	4	5

Mobilization

Please indicate the extent to which you agree or disagree with the following statements:					
	Strongly Disagree	Disagree	Neither Agree Nor Disagree	Agree	Strongly Agree
1. I know how to work with others to solve problems	1	2	3	4	5
2. I have the communication skills to influence people in my community	1	2	3	4	5
3. I know when important community events take place	1	2	3	4	5
4. I **do not** know how to gather information relevant to community issues	1	2	3	4	5
5. I have the skills needed to make improvements in my community	1	2	3	4	5
6. I know how to raise money to do community action projects	1	2	3	4	5
7. I **am not** a leader in my community	1	2	3	4	5
8. I know how to develop leadership in my community	1	2	3	4	5

SOCIAL CONNECTEDNESS QUESTIONNAIRES

Investment in Community Health

In the following section, we list goals that some people think are important for communities in the U.S. In these statements, when we refer to "communities," we mean all communities, not just your own.<br><br>*Should the following be a 'very high priority', 'high priority', 'important but not a top priority', 'low priority', or 'not a priority at all' for communities?*					
	Very High Priority	High Priority	Important But Not a Top Priority	Low Priority	Not a Priority At All
1. Making sure that the disadvantaged have an equal opportunity to be healthy	1	2	3	4	5
2. Making sure that healthy foods are for sale at affordable prices in communities with limited access	1	2	3	4	5
3. Making sure that there are safe, outdoor places to walk and be physically active in communities where there aren't any	1	2	3	4	5
4. Making sure that there is decent housing available for everyone who needs it	1	2	3	4	5
5. Making sure that there are bike lanes, sidewalks for walking and public transportation available so that people do not have to always rely on cars	1	2	3	4	5

Social Cohesion Scale

Please indicate how strongly you agree or disagree with the following statements:					
	Strongly Disagree	Disagree	Neither Agree Nor Disagree	Agree	Strongly Agree
1. People in my community are willing to help their neighbors	1	2	3	4	5
2. People in my community generally get along with each other	1	2	3	4	5
3. People in my community can be trusted	1	2	3	4	5
4. People in my community share the same values	1	2	3	4	5

Social Network Scale

	0	1	2	3 or 4	5-8	9 or more
Considering the people to whom you are related by birth, marriage, adoption, etc...						
1. How many relatives do you see or hear from at least once a month?	0	1	2	3	4	5
2. How many relatives do you feel at ease with that you can talk about private matters?	0	1	2	3	4	5
3. How many relatives do you feel close to such that you could call on them for help?	0	1	2	3	4	5
Considering all of your friends including those who live in your neighborhood...						
4. How many of your friends do you see or hear from at least once a month?	0	1	2	3	4	5
5. How many friends do you feel at ease with that you can talk about private matters?	0	1	2	3	4	5
6. How many friends do you feel close to such that you could call on them for help?	0	1	2	3	4	5
